# Laser Capture Microdissection Revisited as a Tool for Transcriptomic Analysis: Application of an Excel-Based qPCR Preparation Software (PREXCEL-Q)

**Published:** 2009-06

**Authors:** Fatoumata B. Sow, Jack M. Gallup, Randy E. Sacco, Mark R. Ackermann

**Affiliations:** 1 *Department of Veterinary Pathology, College of Veterinary Medicine, Iowa State University, Ames, USA;*; 2 *National Animal Disease Center, Agricultural Research Services, United States Department of Agriculture, Ames, Iowa, USA*

**Keywords:** LCM, laser capture, microdissection, microsection, laser cutting, laser catapulting, PREXCEL-Q, PCR, qPCR, RT, gene expression, real-time PCR, quantitative PCR, qPCR software

## Abstract

The ability to reliably analyze cellular and molecular profiles of normal or diseased tissues is frequently complicated by the inherent heterogeneous nature of tissues. Laser Capture Microdissection (LCM) is an innovative technique that allows the isolation and enrichment of pure subpopulations of cells from tissues under direct microscopic examination. Material obtained by LCM can be used for downstream assays including gene microarrays, western blotting, cDNA library generation and DNA genotyping. We describe a series of LCM protocols for cell collection, RNA extraction and qPCR gene expression analysis. Using reagents we helped develop commercially, we focus on two LCM approaches: laser cutting and laser capture. Reagent calculations have been pre-determined for 10 samples using the new PREXCEL-Q assay development and project management software. One can expect the entire procedure for laser cutting coupled to qPCR to take approximately 12.5-15 h, and laser capture coupled to qPCR to take approximately 13.5-17.5 h.

## INTRODUCTION

Relatively recent advances in science including PCR and LCM have allowed the rapid analysis of normal and diseased tissues at the DNA, RNA and protein levels. The etiology and pathology of many diseases can be linked to alterations in genes, gene products, and the signaling pathways stimulated by these gene products. The molecular alterations observed may be specific to a cell type, and attempts at correlating such defects to the cells in question can be hampered by the cellular heterogeneity of tissues. This can limit the meaning of biological data obtained since the many different cell types which comprise a certain tissue may not be represented individually by analysis of and information from whole-tissue. Further, the use of whole-tissues renders it impossible to determine which cellular constituents contribute to the expressed gene signal of interest, especially since a two-fold signal difference is considered significant in gene expression studies. In addition, proteomic and genomic techniques rely heavily on the procurement of homogeneous cell populations. While cell cultures and cell lines are frequently used to study pure cellular populations in a controlled milieu, the genetic information obtained *in vitro* may not necessarily represent the molecular events occurring in the actual tissue environment. Detailed molecular and biochemical analyses of *in vivo* interactions require the ability to analyze specific cell populations within their heterogeneous tissue environment.

LCM (1-4) is a recently developed technology that provides the means to isolate or enrich single cell types or unique cellular structures from heterogeneous tissues while preserving the original tissue’s morphology and without introducing contamination from surrounding cells. As its name implies, the LCM technique is based on the use of a near infra-red laser with pinpoint precision fitted to an inverted microscope. The principle steps of LCM have an elegant simplicity: a tissue sample is mounted on a slide, and cells of interest are visualized (morphologically, or based on the use of a marker specific to the cell type). A transparent 100 μm-thick ethylene-vinyl acetate film coated on a cap is then placed over the tissue section by pulling the cap holder (loaded with a cap) over the tissue, then lowering the cap film-side down onto, and in direct contact with the tissue. The diameter of the laser beam can be adjusted from 7.5 to 30 μm, depending on the size of the cell or group of cells one wishes to select. The low-energy laser, administered in pulses, causes the thermoplastic film to melt, bind to, and lift the targeted cells out of the tissue. No damage occurs to the biological macromolecules collected as the energy coming from the laser is absorbed completely by the film, and the pulsing of the laser is only performed for milliseconds. All unwanted cells are left behind in the original tissue, which could be further dissected if needed, provided the tissue is well preserved. The samples captured by LCM can be immediately harvested for molecular analyses. This technique is very well suited for the isolation of single cells or small groups of cells. The Arcturus PixCell II Laser Microdissection apparatus is an excellent system for isolating cells of interest by laser capture.

Recently, a new generation of microdissection apparatuses has been unveiled: laser cutting (MMI, Leica), laser catapulting (PALM), and scanning laser microdissection (XMD) (5) systems. The PALM microlaser/microbeam systems are based on the ability of the laser to microdissect tissues and to pressure-catapult the collected cells into a collection or resuspension-lysis buffer. This is often referred to as a precise “non-contact” laser pulse system. Here, an ultra-violet (UV-A) laser with a beam spot of less than 1 μm in diameter is used to cut selected cells. After microdissection, the cells are catapulted directly (against gravity) into the lid of a Zeiss PALM 0.5 ml microfuge tube, which minimizes contamination from neighboring tissue and eliminates the possibility of contamination by way of direct mechanical contact with the source sample from which selected regions or cells are being taken.

### Diversity of applications of LCM

The LCM technology has been used widely in cancer research, therapeutic efficacy studies, forensics, drug interactions, and toxicity assessments. In studies involving host and pathogen interactions, it is possible to identify the first cells targeted by invading pathogens, differentiate infected cells from non-infected cells, and examine the pattern of viral or bacterial distribution. In studies involving drug interactions and therapeutic efficacy, it is possible to determine where the drug goes, how it affects safety and efficacy in tissues, how cells respond to treatment by comparing whole tissue to a specific structure of the tissue, and even identify critical safety biomarkers. Protein studies on LCM-derived cellular material can be performed as well. Although this protocol summarizes an LCM-based approach to study gene expression by qPCR in ovine macrophages, it can be adapted to study any animal cell type. LCM for plant material is not addressed in this protocol, but, with appropriate use of other fixatives preceding IHC, and proper adjustments to laser power strength and duration, any plant cell of interest can be similarly accessed.

### Limitations of the LCM and LCM-qPCR techniques

There are several drawbacks associated with LCM. Some of these relate to sampling issues, such as the stability of the isolated material (e.g. RNA degradation) and to the quantity of material. Frequently, it may be necessary to pool material from multiple slides/tissues to get enough samples for downstream analyses. Probably the most obvious limitations of LCM are those associated with it being a microscope-based technology. Since LCM scopes are inverted by design, they must focus first through the glass slide itself before they obtain the tissue or cell image of interest. In addition, it is impractical in LCM to coverslip sections, thus recognizing tissue morphology is often more difficult. In some cases, LCM can be coupled to immunofluorescence microscopy (as in this study), but such assays rely on the availability of antibodies specific to desired cellular markers. Other microscopy-based concerns relate to the inability to use certain difficult-to-section tissues (e.g. bone or mineralized tissue, tooth pulp, some plant material) as well as to the strength or weakness of tissue adherence to the slides in the first place (e.g. too strong, and collection can be impossible, too weak, and the subject tissue may fall off of the slides entirely, before LCM can be performed). In addition, LCM-collected cells can be contaminated by material adjacent to the subjects of interest if the tissue is not suitably adherent to the chemically-treated glass substrate used.

There is also a current need to improve and/or come up with more consistent global profiling methods using LCM-coupled qPCR and qPCR in general. Recent concerns have been voiced as to the rare use of LCM and lack of awareness about qPCR inhibition (Bustin, S. How Reliable is Your qPCR Data? Drug Discovery & Development, March 01, 2006, http://www.dddmag.com/reliability-of-qPCR-data.aspx). qPCR also requires extensive calculations, standard curves, and optimal reaction efficiencies. Attention to these details takes time and can limit investigators to assessment of one or just a few genes. If inhibition is not eliminated, or if calculations and optimizations are not performed correctly, qPCR will yet generate results; however, they can be very inaccurate. This is especially true for LCM-qPCR because the sample size is very tiny and therefore, mistakes become magnified. Unfortunately, investigators can be completely unaware that their data is faulty. To address many of the concerns above, we have invented PREXCEL-Q (P-Q) (6, 7), a program that allows swift calculations of reagent and sample needs for every aspect of qPCR. In addition, the program has a built-in function specifically tailored for LCM-qPCR on which we have published previously (8, 9) and have further optimized for lung macrophage studies.

### P-Q

The P-Q program (7) has been a very helpful implement in addressing the common time-consuming perfunctory concerns with qPCR setups by automatically calculating amounts of all needed reagents (primers, probes, master mix), gauging total sample material needed, assisting directly with appropriate standard curve designs, identifying the dynamic dilution range of sample material within which qPCR inhibition is absent and target amplification efficiencies are highest, automatically generating drawings and printouts summarizing reaction formulations, and estimating total cost of the assay. P-Q has also been designed to address parameters related specifically to LCM, such as pg of nucleic acid per cell-type, number of cells collected per sample, volume of each final collected sample and the most conservative use of the collected material in creating standard curves are all calculated by the program.

### MIQE and the RDML Consortium

Finally, it is important to remind all current users of qPCR technology, and all publishers of qPCR data, to follow the responsible guidelines set forth by MIQE (or MIqPCR): Minimum Information for Publication of Quantitative Real-Time PCR Experiments, which is part of the Real-time PCR Data Markup Language (RDML) Consortium created by Stephen Bustin and presently tended to by Vladimir Benes, Jeremy Garson, Jan Hellemans, Jim Huggett, Mikael Kubista, Reinhold Mueller, Tania Nolan, Michael Pfaffl, Gregory Shipley, Jo Vandesompele, Carl Wittwer, Steve Lefever, Andreas Untergasser and Filip Pattyn. The aim of MIQE, which is coordinated under the umbrella of MIBBI (Minimum Information for Biological and Biomedical Investigations) is to provide authors, reviewers and editors specifications for the minimum information that must be reported for a qPCR experiment in order to ensure its relevance, accuracy, correct interpretation and repeatability. A checklist, which should be submitted along with the paper, is available for authors in preparing a manuscript employing qPCR. This organization and its philosophy were created in effort to standardize the way people perform and report their qPCR studies (http://medgen.ugent.be/rdml/guidelines.php). Following these guidelines will encourage better experimental practice, allowing more reliable and unequivocal interpretation of quantitative PCR results (http://www.sabustin.org/). The RDML Consortium’s aim is to encourage and foster the use of a universal qPCR data exchange format. The RDML format will facilitate the exchange of data between instruments and data analysis software, between different users and even allow submitting qPCR data to central repositories or as supplemental data to a paper. MIQE (MIqPCR) guidelines have been developed by Stephen Bustin in collaboration with MIBBI to assure that data files contain the minimal information allowing unambiguous interpretation of the data. The central focus of this stalwart endeavor is to create a universal qPCR data format that can be used by anyone regardless of qPCR instrument and analysis software and to supplement this format with guidelines and tools to achieve maximum benefits with minimal burden for users. Founders of this organization define its members as “anyone who helps developing, makes suggestions and comments or just declares support for the RDML initiative.” Further information on the RDML Consortium can be found in the following websites: info@rdml.org, http://www.rdml.org, http://sourceforge.net/projects/rdml/.

### Alternative methods

Flow cytometry can be used to isolate cells of interest from a suspension, but it relies on the use of a specific cellular marker for selection, and commonly requires enzymatic digestion or other treatments to isolate of cells from solid tissue samples. Histopaque gradient methods for isolating different leukocyte populations can also be met with difficulties when contaminating platelets are carried over into the final samples, and when centrifuge speeds, temperature of material and tube-type usage have not been optimized. Similar problems are found using Percoll gradients to isolate tissue leukocytes (e.g. gut lymphocytes (IELs, LPLs)). Additional concerns with histopaque and Percoll isolations include limited quantities of blood or tissue samples, low target cell population(s), and cost. Thus, LCM remains the best procedure for the isolation and enrichment of specific cells from samples immobilized on a solid matrix.

Recent responses obtained from a MIQE questionnaire revealed that only 12% of scientists performing qPCR on a regular basis use LCM technology (Bustin, S. How Reliable is Your qPCR Data? Drug Discovery & Development, March 01, 2006, http://www.dddmag.com/reliability-of-qPCR-data.aspx). In addition, qPCR coupled with LCM might be considered too daunting to undertake, as both techniques are often rife with complexities not often surmounted by experienced investigators and novices alike. We offer here a fail-safe approach to LCM-coupled qPCR in the hope to increase its usership – in keeping with the wishes of leaders in the qPCR field (10). In revisiting this important technique, we have found several ways to improve its user-friendliness and reliability: 1) use of the P-Q program to facilitate qPCR setups; 2) use of a defined kit that incorporates reagents for both LCM RNA extraction and subsequent qPCR (which we recently co-developed with Invitrogen) and a correlate master mix kit used for genomic DNA contamination analysis in all sample isolates; 3) use of the two particular master mixes mentioned in this study is convenient in that they can both use the same thermocycling program, and can therefore be used simultaneously on the same plate; 4) demonstration of using LCM on a difficult tissue, such as lung, from which epithelial cells (8, 9) and macrophages can be collected. For simplicity’s sake, in this manuscript, we show the reagents required for performing LCM-qPCR on 10 samples throughout.

### Conclusions

Since its discovery in 1997, the LCM technique has undergone many refinements and helpful modifications (2-4). The analysis of purified cells populations from selected regions of a tissue can now be automatically performed by the use of a computer-controlled stage combined with the LCM microscope. Improvements in DNA and RNA extraction procedures have allowed the increased yield and purity of isolated material for downstream molecular analysis. It is also possible to collect enough sample material for protein isolation, profiling, and discovery. With our current approach, we can repeatedly and reliably analyze basal and induced gene expression of cells obtained from diseased animals (respiratory syncytial virus, RSV, for example (11)) for comparison to control animals, thus adding a powerful dimension to the study of host factors regulating disease progression. The present protocol represents a tightly-defined approach to LCM-coupled qPCR, one which should be routinely implemented and advocated as a standard operating procedure.

## EXPERIMENTAL DESIGN

### Materials

Reagents:

- Lambs ♦ **CRITICAL STEP** Animal use and all experimental procedures were approved by Iowa State University’s Animal Care and Use Committee. Be sure to obtain appropriate national and institutional approval prior to performing experiments using human and/or animal subjects;
- Specimens for analysis (frozen-tissue sections, paraffin-embedded sections);
- Disposable cryomolds (Surgipath Medical Industries Richmond, IL; Cat. No. 03040);
- Cryopreservation solution such as OCT (Tissue-Tek OCT, VWR International, Batavia, IL; Cat. No. 25608-930);
- Acetone (Sigma, St. Louis, MO) **! CAUTION** Acetone liquid and vapor are extremely flammable and can cause eye and respiratory tract irritation. Wear appropriate personal protective equipment (PPE) such as gloves, lab coat, safety goggles, and respirator. Also use with proper ventilation, such as within confinements of a chemical hood;
- Stock TRIS (good for 1 year): 60.57 g TRIS base in 500 ml H_2_O. Add 30 ml concentrated hydrochloric acid (HCl). Adjust final pH to 7.6 with HCl. Bring total volume up to 1 liter with H_2_O **! CAUTION** HCl is poisonous and corrosive; liquid and mist inhalation can cause severe burns and may be fatal is swallowed or inhaled. Use proper PPE protection measures and a ventilation system while using HCl;
- Working TRIS buffer: Dilute stock TRIS 1:10 in nuclease-free H_2_O;
- Normal goat serum (Sigma, St. Louis, MO);
- PBS tablets (Sigma);
- Nuclease-free PBS, pH 7.4 (can be made with nuclease-free H_2_O and PBS tablets);
- TRIS/PBS: 10 ml TRIS working solution and 90 ml PBS (0.01M) in nuclease-free H_2_O;
- Nuclease-free H_2_O;
- Bovine serum albumin (BSA; IgG free, protease-free; Jackson Immunoresearch Laboratories, West Grove, PA; Cat. No. 001-000-162);
- TRIS/PBS + 3% BSA: 3 g BSA in 100 ml TRIS/PBS diluting buffer;
- Antibodies:
- 1) Mouse anti-bovine CD68 (Dako, Carpinteria, CA; clone KP1 Cat. No. M0814) or mouse anti-bovine CD11b (AbD Serotec, Raleigh, NC; Cat. No. MCA1425G) for detection of macrophages;
- 2) Affinity purified antibody biotin labeled goat-anti mouse IgG (Kirkegaard-Perry Labs, Gaithersburg, MD; Cat. No. 16-18-06);
- 3) Cy3 conjugated streptavidin (Rockland Inc., Gilbertsville, PA; Cat. No. S0000-04);
- 4) Alexa Fluor 488 F(ab’)_2_ fragment of goat anti-mouse IgG (Invitrogen, Carlsbad, CA; Cat. No.A11017);
- Hematoxylin: Shandon Lipshaw acidified hematoxylin solution (Cat. No. 6765004). Dilute Shandon’s acidified (pH 5.2) hematoxylin 1:3 with deionized H_2_O (d.i. H_2_O). **! CAUTION** Wear gloves when handling, as hematoxylin is a contact hazard; Also harmful when swallowed;
- Scott’s tap water: 10 g MgSO_4_ and 2 g NaHCO_3_ in 1 liter d.i. H_2_O;
- 100% ethanol (ethyl alcohol, absolute, 200 proof for molecular biology; Sigma Cat. No. E7023). Use this 100% ethanol and the nuclease-free H_2_O to prepare 95%, 75%, and 50% ethanol (vol/vol). **! CAUTION** Wear proper PPE when handling. Do not ingest. This is a flammable, and a contact hazard;
- Xylene (Mallinckrodt Baker) **! CAUTION** Use within the confinements of a chemical hood with appropriate ventilation, as vapors from this agent are harmful and fatal. Also wear gloves when handling this agent;
- CellsDirect™ One-Step qRT-PCR Kits with 1 M free ROX already in the master mix (which we co-developed in 2005 with Invitrogen; Cat. No. 11754-100 or Cat. No. 11754-500). Note: other commercial RNA extraction and qPCR master mix kits are available, however we recommend this particular kit as it has worked extremely well in our particular LCM applications because of its recent inclusion of the very high-fidelity reverse transcriptase (RT) enzyme, SuperScript™ III. CellsDirect™ One-Step qRT-PCR Kits with ROX provided in a separate tube (for machines that cannot tolerate 500 nM final [ROX], are also provided as Cat. No. 11753-100 or Cat. No. 11753-500). Components of the Cat. No. 11754 kit are listed in Table 1;
- Platinum Quantitative PCR SuperMix-UDG with ROX (Invitrogen, Cat. No. 11743-100) for assessing the presence of genomic DNA (gDNA) contamination in RNA isolates;
- Primers and probes: see Table 2.

**Table 1:** qPCR master mix kit components

	CellsDirect™ One-Step qRT-PCR mix with ROX Kit Components Invitrogen Catalog nos. 11754-100 and 11754-500	

Kit Size	100 rxns	500 rxns
Resuspension Buffer	10 ml	10 ml
Lysis Enhancer	1 ml	1 ml
DNase I, Amplification Grade (1 U/μl)	500 μl	2 × 1.25 ml
10X DNase I Buffer	160 μl	800 μl
20 mM EDTA	400 μl	2 × 1 ml
SuperScript™ III RT/Platinum^®^ Taq Mix (with RNaseOUT™ Ribonuclease Inhibitor	100 μl	200 μl
2X Reaction Mix w/ROX	2 × 1.25 ml	12.5 ml
50 mM MgSO_4_	1 ml	1 ml
DEPC-treated water	2 ml	12.5 ml
HeLa Total RNA (10 ng/μl)	10 μl	10 μl

**Table 2:** List of primers/probes used for real-time qPCR

Primer name	Sequence (5’-3’)	GenBank Accession number or source

MCP-1 forward	GCTGTGATTTTCAAGACCATCCT	Based on conserved sequences reported elsewhere (15)
MCP-1 reverse	GGCGTCCTGGACCCATTT	
MCP-1 probe	FAM-AAAGAGTTTTGTGCAGACCCCAACC-TAMRA	
TLR4 forward	CCATCGCCGCCAATATCA	DQ922636
TLR4 reverse	TGGGACACCACGACAATAACC	
TLR4 probe	FAM-CCAGGAGGGTTTCCACAAAAGCCGT-TAMRA	
IL-6 forward	GCTGCTCCTGGTGATGACTTC	NM_001009392
IL-6 reverse	GGTGGTGTCATTTTTGAAATCTTCT	
IL-6 probe	FAM-CTTTCCCTACCCCGGGTCCCCTG-MBGNFQ	
TLR3 forward	TGTTTGCGAAGAGGGATGTTT	AM981301
TLR3 reverse	AAGCATTTACCCGTTCTTTCTGA	
TLR3 probe	6FAM-AATCTCATTGCATCTTGAATTGGCCGG-TAMRA	
TLR7 forward	GCAGCCTGTTCTGGAAAATCTT	AM981305
TLR7 reverse	TTTGCGTACTTGTCTGTCATCACA	
TLR7 probe	FAM-CCCAGAGCATACAGCTTAGCAAAAAGACAGTG-TAMRA	
TLR8 forward	TGTCACGGACTGGGTGATCA	AM981306
TLR8 reverse	GCACGTTCTTGTCCTCACTCTCT	
TLR8 probe	FAM-TGAATTGCGCTTCCACCTGG-TAMRA	
hRSV forward	GCTCTTAGCAAAGTCAAGTTGAATGA	M11486 (11)
hRSV reverse	TGCTCCGTTGGATGGTGTATT	
hRSV probe	FAM-ACACTCAACAAAGATCAACTTCTGTCATCCAGC-TAMRA	
RPS15 forward	CGAGATGGTGGGCAGCAT	Reported in (6, 13)
RPS15 reverse	GCTTGATTTCCACCTGGTTGA	
RPS15 probe	VIC-CCGGCGTCTACAACGGCAAGACC-TAMRA	

### Reagent Setup

**Primer and probe design.** We designed all primers and probes mentioned in this work using Primer Express version 2.0 (Applied Biosystems). Primers and probes designed in this fashion require an adjustment of the final qPCR reaction Mg^+2^ concentration to 5.5 mM. **! CAUTION** Be aware that different primer-probe designing programs generate primers and probes with different ionic strength requirements of the final master mix in order to attain the intended (program-calculated) melting temperature (T_m_) value of each oligo. Be certain to use the recommended Mg^+2^ concentration calculated or suggested by the particular software and/or particular master mix you decide to use. Detailed accounts of these and other important considerations that accompany good primer and probe design can be found elsewhere (8).

**Master mix considerations.** Two avenues are available when choosing a master mix to use for qPCR. Although we speak only of using a “one-step” qPCR master mix (which contains both RT and Taq enzymes) in this study, a “two-step” format (which uses a qPCR master mix that does not contain RT enzyme, only a variety of Taq) is also available. By definition, a “two-step” qPCR procedure, implies that RNA has been converted to cDNA in a separate step preceding qPCR. LCM-derived RNA samples can be converted to cDNA (with or without linear amplification) and stored at -80°C for several years. Next, the cDNA can be subjected to qPCR using an appropriate master mix (without RT enzyme) to complete the qPCR – the same kind of master mix can also be used in the event that your LCM-derived samples are genomic or organismal DNA as the template is already “DNA.”

**Figure 1:**
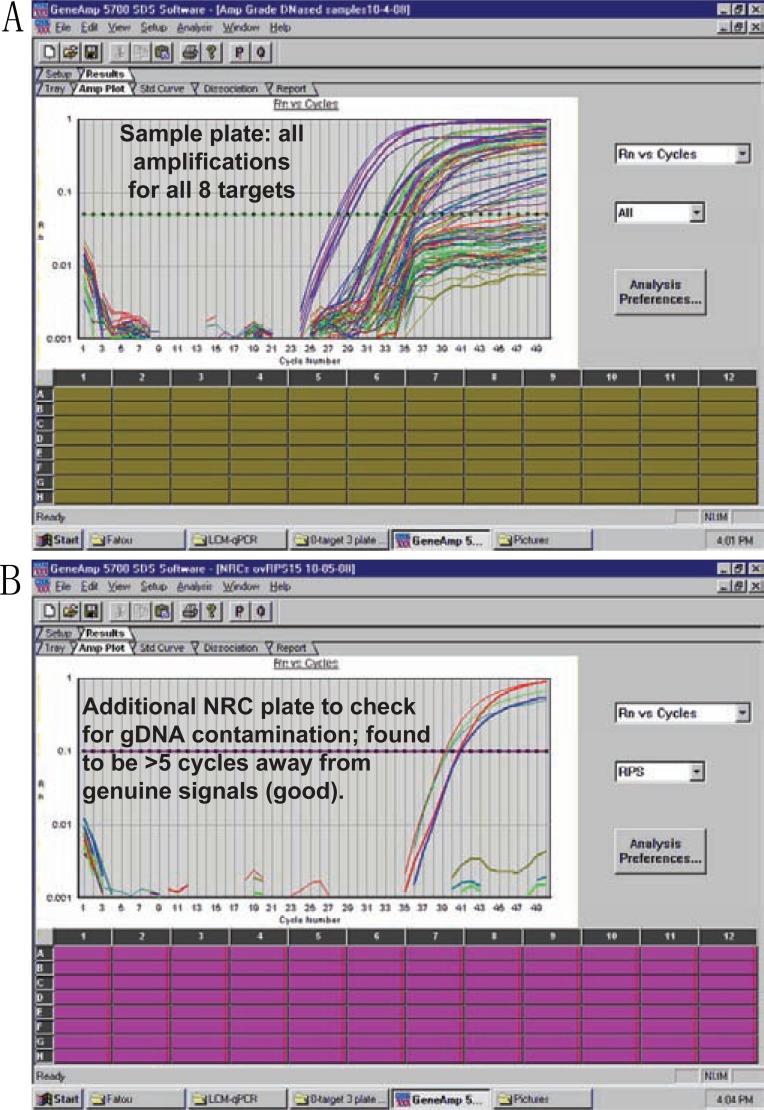
Typical LCM-qPCR Amplifications. A, Typical qPCR amplifications from our LCM samples; B, NRC sample (genomic DNA) amplifications.

**Testing for genomic DNA contamination in LCM-derived RNA samples.** Even though proper use of the CellsDirect™ One-Step qRT-PCR Kit with ROX includes a DNase-treatment step of LCM-RNA isolates, carryover DNA can still be present. In order to be certain that signals generated by qPCR are truly resultant of transcriptomic (mRNA) as opposed to genomic nucleic acid (gDNA) amplifications, it is imperative to include sample wells that contain master mix with no RT enzyme. Such reactions are called “no RT control” reactions (NRC), wherein only contaminating gDNA (if present) will be amplified and thus serve as an indicator as to what degree the RNA samples are gDNA-free. In our studies, we have found it most advantageous to use a highly expressed gene such as a reference gene (formerly known as a “housekeeping gene”) for this assessment. We used ovine ribosomal protein S15 (ovRPS15) as the reference gene here since a nearly homogeneous cell population was being studied. Since reference genes are normally abundant in samples and/or tissues, they should provide the best chance of finding any indication of gDNA presence. gDNA signal does not contribute significantly to genuine sample signals if a difference in Ct values of greater than 5 (between the NRC master mix and the sample master mix) is observed. gDNA contribution to genuine transcriptomic signal can be mathematically obtained as follows: 1/E_AMP_^(NRC Ct – Sample Ct)^, where “E_AMP_” = Exponential amplification = 10 ^-1/m^, m = slope of target standard curve, “Ct” = threshold cycle, and “NRC” = no RT control (we use a reference gene as qPCR target for this). Contaminating gDNA qPCR signals 5 or more cycles away from genuine *transcript-generated* qPCR signals have a nearly negligible impact on final qPCR results, e.g. 1/(E_AMP_ of 2)^5^ = ~3.13% signal contribution when E = 100% (Fig. 1).

In our experience with non-LCM-based qPCR, using Turbo DNase (Ambion/ABI), contaminating gDNA signals have always been greater than 12 cycles (but typically 13 cycles or more) away from our genuine transcriptomic target qPCR signals, indicating to us that gDNA-related contributions to each of our genuine/intended one-step qPCR target amplifications have been clearly minimized (<1/2^12^ = ~0.024% signal contribution when E = 100%, where E = E_AMP_ - 1). Realize, however, that these contributions compound upon themselves when both target and reference gene signals are affected by contaminating signal contributions, so, knowing the extent of this contribution is absolutely necessary in any experiment using a reference gene to quantify gene expression. NRC reactions are always necessary to prove that your LCM-qPCR Ct signals are not merely the result of amplified gDNA in cases where the chosen primers or probes do not span a genomic intron^6^. In the present study, we used Amplification Grade DNase I (as provided in the Invitrogen kit used for one-step qPCR) for the DNase treatment of our LCM samples.

**Reference gene considerations.** Although the use of reference (housekeeping) genes has come under fire over recent years due to their variable expression in many tissue and cell samples, qPCR performed on RNA from homogeneous cell populations collected by LCM is perhaps the best candidate for their use in normalizing qPCR gene expression data. Assessment of reference genes in total RNA from whole tissue samples is inherently subject to the differential contributions of reference gene transcripts from a myriad of cell types - thereby rendering useless the advantage initially sought by using a reference gene in the first place. LCM-collected homogeneous cell samples offer an intuitively better paradigm wherein the use of a reference gene for normalization purposes is well-suited. However, any normal cell, when compared to its neoplastic incarnation, runs the same risk of variable reference gene expression and thereby, caution is again advised when using a reference gene to normalize gene expression data gathered from two similar but metabolically different cell types (12). In this manuscript, we have chosen to use the reference gene, ovRPS15 as it has demonstrated great stability in many of our particular qPCR studies for several years now, with the exception of studies involving qPCR analyses of total RNA extracted from tissues of animals at different stages of ontogeny. Although we use only one reference gene here, other investigators choose to test up to 10 reference genes until they find one, two or three that are stable in their particular system. SABiosciences offers an excellent platform in which to test multiple reference genes in mouse, rat and human qPCR metabolic pathway studies. Be certain to test whatever reference gene (or genes) you choose for stability in your particular system. The ideal reference gene should generate the same Ct value in all samples when all samples are loaded into the qPCR reactions equally.

**Figure 2:**
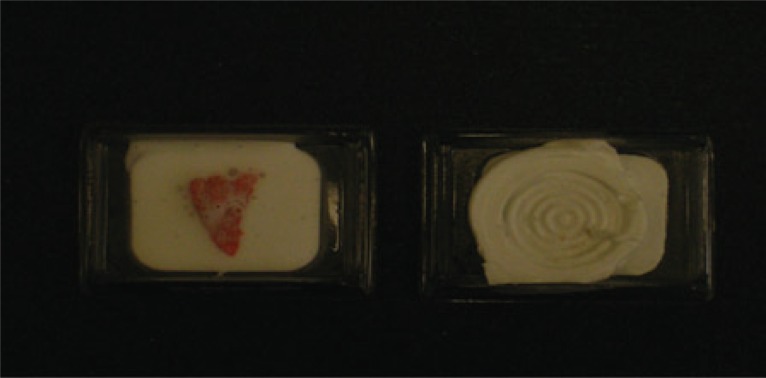
Excised tissues frozen in cryomolds containing OCT compound. Freshly-necropsied lung tissues (the example in this paper is sheep lung) are placed into plastic disposable cryomolds containing OCT. Top and bottom sides of the block are shown.

### Equipment

- Gloves;
- Pipets;
- Pipet tips (barrier filtered, certified nuclease-free);
- Glass slides (pre-cleaned, positively-charged and poly-L-lysine coated AAPS 25 × 75 × 1 mm);
- Slide rack;
- Slide box;
- Slide containers (5-slide mailers; Coplin jars; metal (30-) slide rack; ProHisto, LLC staining chambers);
- Cryostat (e.g. Leica CM 1900);
-80°C freezer;
- 4°C fridge or cold room;
- PAP-pen (liquid wax pen; BioGenex, San Ramon, CA) (optional);
- CapSure HS LCM Caps (LCM 0214 MDS Analytical Technologies) for laser capture
- Prep Strip™ (Arcturus/Molecular Devices, Cat No. 11179-00);
- 0.5 ml PALM sample capture tubes for laser cutting/catapulting (Zeiss; LMPC set D Cat. No. 415101-4402-000);
- 0.5 ml MicroAmp tubes for laser capture (Gene-Amp Tube, Applied Biosystems/Ambion; Cat. No. N8010611) for laser cutting (no need for glycerol or oil in the caps; the direct use of 44 μl lysis buffer works great, provided you cut cells in a timely manner to avoid drying of the buffer in the cap). ♦ **CRITICAL STEP** Be sure the 44 μl lysis buffer is kept at 4°C prior to its application to the inside of the tube lid;
- 1.5 ml nuclease-free microfuge tubes (e.g. MidSci microstein St. Louis, MO; Microstein™ “no more sore thumbs” 1.5 ml microfuge tubes; Cat. No. MIC1004);
- Heating blocks set to the following temperatures: 37°C, 50°C, 70°C, 75°C, 95°C **♦ CRITICAL STEP** It is important to have multiple heating blocks already set up at the temperatures to avoid delays during experimental procedure. Also, it is important to fill all heating block wells half way with water to ensure optimal heat transfer from block to tubes;
- Ice bucket (to keep tubes containing LCM caps or tubes just removed from PALM stage area after cutting of cells);
- Vortexer;
- Dessicator for slides (optional);
- Chemical hood with proper ventilation;
- Laser-capture microdissection apparatus (e.g. Pix-Cell II from Arcturus/Molecular Devices or PALM from Zeiss). **! CAUTION** Although this manuscript goes into great detail on a step-by-step basis regarding the LCM technique and the use of two types of LCM scopes, we do not recommend using this paper as a substitute for required LCM training offered by your particular institution;
- qPCR plates or tubes depending on platform used (we use white-welled 96-well plates from Eppendorf (Cat. No. E951022043) or normal frosted 96-well plates from ABI (Cat. No. 403012));
- Quantitative PCR machine (e.g. GeneAMP 5700 used in this study. Others: GeneAmp 7300, 7500, 7900; Stratagene Mx3005P, Mx4000; Roche LightCycler 480, etc.). **! CAUTION** Be aware of the idiosyncrasies associated with each different qPCR machine platform, e.g. due to differences in optic technology and photomultiplier tube sensitivity from machine to machine, proper use of internal reference dyes such as ROX must be adjusted accordingly (see also Bustin, Nature Protocols 2006);
- Primer/probe design software (e.g. Primer Express version 2.0 from Applied Biosystems).

### LCM-qPCR Procedure

#### 1) Procurement of tissue (? TROUBLESHOOTING see Table 3)

**(A) Tissue collection and storage ♦ TIMING 2 h**

Freshly-necropsied lung tissues (the example in this paper is sheep lung) are placed into plastic disposable cryomolds containing OCT. More OCT is applied over the tissues, and these are then placed onto blocks of dry ice until the tissues embedded in OCT are frozen to a solid white (Fig. 2). All samples are then transferred immediately to -80°C for storage. **♦ PAUSE POINT** Samples are stable for years in this state.

**(B) Tissue sectioning (per 5 sections, assessed in duplicates for a total of 10 samples) ♦ TIMING 30 min-1 h**

**Figure 3:**
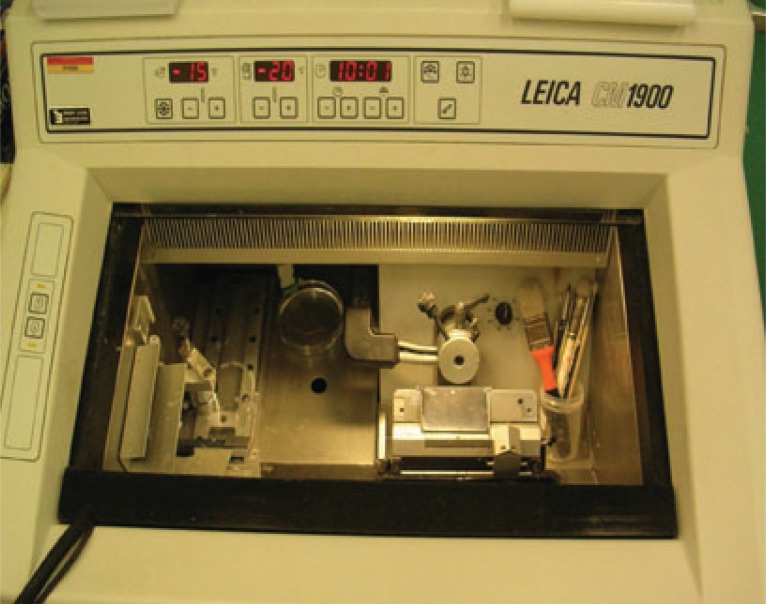
The Leica CM1900 Cryostat. This apparatus is used to section frozen tissue onto slides.

**Figure 4:**
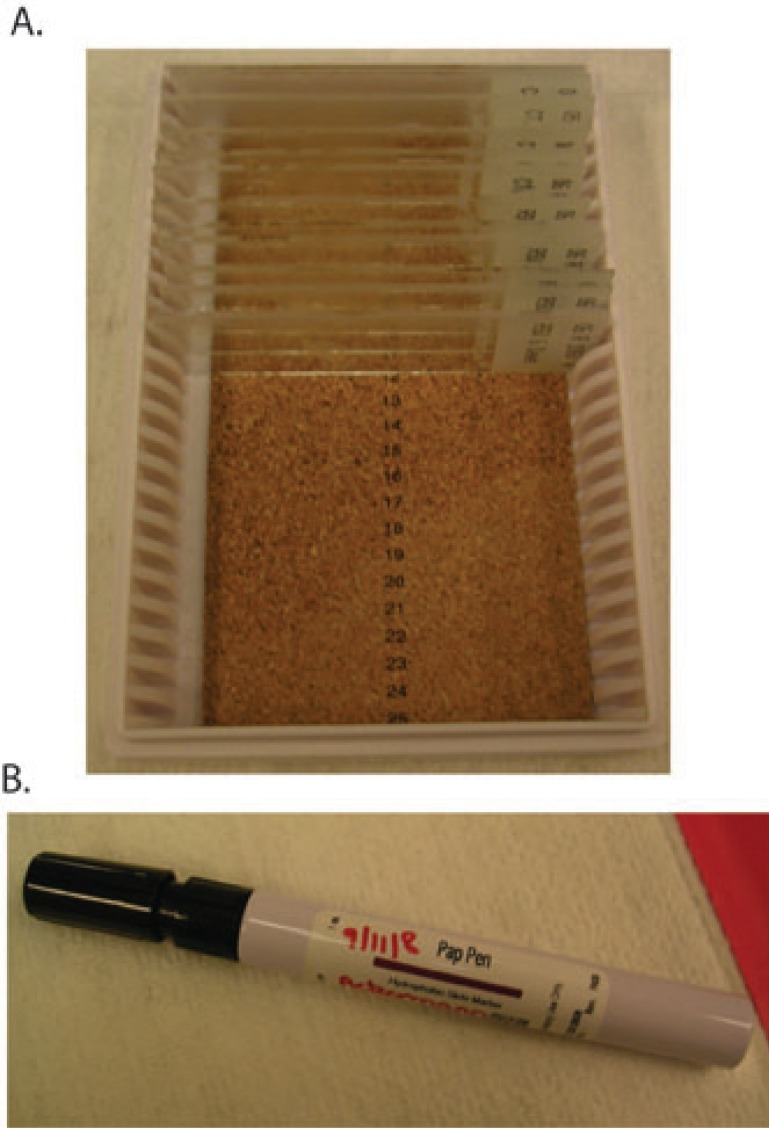
Slide storage box and PAP-pen. A, After sectioning of tissues, slides are stored in a custom slide box typically found in many histology labs; B, The PAP-pen is a liquid wax pen which is used to demarcate a region above and below the tissue on each slide to keep reagents from bleeding off during IF.

- OCT-embedded frozen tissues are cut at -25°C with a cryostat (Fig. 3) into 5 μm LCM sections, and placed onto pre-cleaned glass slides. This procedure is considered a standard Leica microtome procedure (see: http://www.hbu.de/range.htm). **♦ PAUSE POINT** Frozen sections: for RNA-based applications we have found that frozen sections are stable at -80°C for up to 7 days. **! CAUTION** We previously observed a dramatic decrease in qPCR signals when slides stored longer than 7 days were used in the LCM-qPCR application (13). In accordance with the Molecular Devices (Molecular Devices, Mountain View, CA) protocol suggestions, we did not dry the sections after cutting, but instead stored them (directly after cutting onto slides) first at -20°C in a box (Fig. 4A) inside the cryostat itself, then in acetone for 5 min;
- LCM can be used on paraffin-embedded tissues in which case other protocols are available (Molecular Devices protocol online http://www.moleculardevices.com/pages/reagents/paradise.html).

**(C) Slide preparation (per 5 slides, assessed in duplicates for a total of 10 samples) ♦ TIMING 2 min**

- Next, a small region above and below the section is dried off with a clean napkin, and a PAP-pen (Fig. 4B) is used to demarcate a region below and above the tissue section;
- It is important to draw the lines at a safe distance from the tissue and to avoid touching the tissue with the liquid wax, as it is not certified nuclease-free. If multiple slides are being used, label them appropriately.

#### 2) Immunofluorescence (IF) staining of leukocyte populations in frozen tissue sections (? TROUBLESHOOTING see Table 3)

**(A) Fixation and blocking**

**Figure 5:**
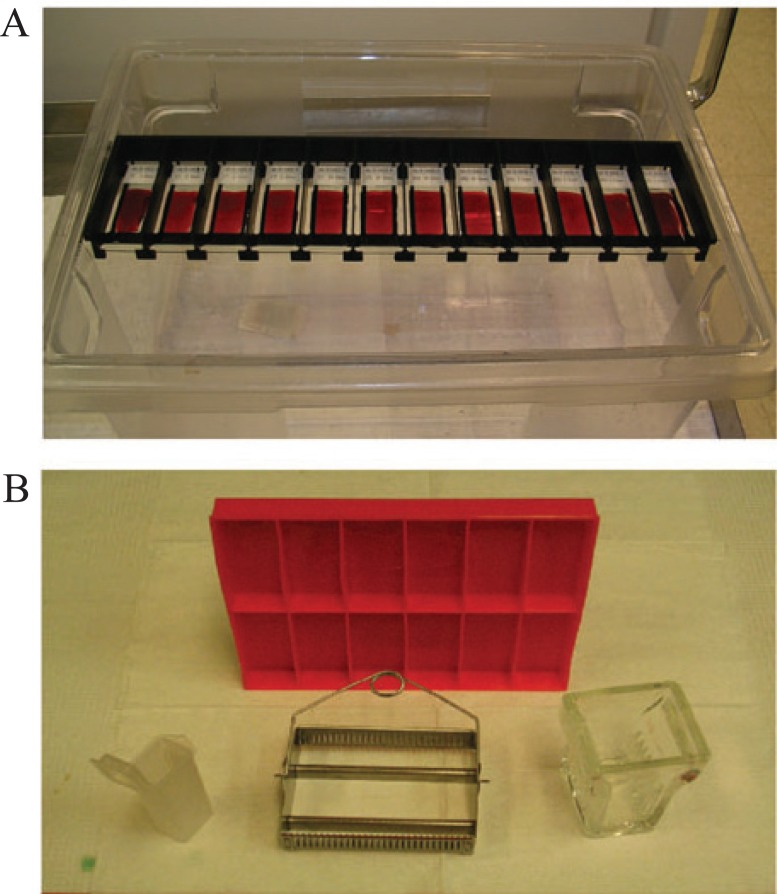
Slide holders for staining and storage. A, Metal slide rack positioned atop a humidifying chamber is used during a staining procedure (such as hematoxylin staining); B, Types of containers that can be used to process and handle slides.

All the following steps are performed at 4°C on a slide rack or slide-mailers (Fig. 5A and Fig. 5B), unless otherwise specified: IF ♦ **TIMING 42 min**.

- Fix cells in acetone for 5 min room temperature;
- Frozen tissue sections are rehydrated in 1X TRIS buffer for 5 min, and loaded into ProHisto, LLC staining chambers;
- Block with 5% normal goat serum in 1X PBS for 30 min at RT;
- Wash with 1X TRIS buffer for 2 min.

**(B) Staining procedure**

The following steps can be performed using Option A or Option B depending on which secondary conjugates are available or desired:

First option ♦ **TIMING 45 min** (hands-on) – but includes a 16-24 h incubation

- Add primary antibody to slides placed in staining chambers (we typically use primary antibody at 1:400 in TRIS/PBS + 3% BSA). Incubate overnight (16-24 h) at 4°C;
- Remove primary antibody solution. Rinse slides with TRIS buffer for 2 min;
- Place slides in rack. Add 400 μl diluted secondary antibody (Alexa 488 antibody) or avidin-enzyme conjugate (if using biotinylated primary Ab). Incubate 30 min at 37°C;
- Rinse with 1X TRIS buffer for 2 min;
- Rinse in d.i. H_2_O for 5 min. Remove from staining chamber. Sections can be counterstained with hematoxylin as described below.

Second option ♦ **TIMING 2 h 21 min**

- Incubate sections with primary antibody (or antibody directly conjugated to a fluorescent dye) by making a 1:1 mixture of the primary antibody (e.g. mouse anti-bovine CD68) with biotinylated goat anti-mouse IgG, and incubate this antibody mixture at 37°C for 1 h, then add a diluted amount of this antibody mixture to the sections for 1 h at 4°C
- Wash slides three times for 2 min each with PBS + 0.1% Tween, then add streptavidin-Cy3 (diluted 1:500) for 10 min at 4°C
- Wash the slides with 4°C PBS for 5 min, and continue with the hematoxylin staining procedure below

#### 3) Hematoxylin staining ♦ TIMING 7 min (? TROUBLESHOOTING see Table 3)

**(A) Hematoxylin staining and dehydration of the tissue**

The procedure can be carried out on slide racks as follows:

- nuclease-free 75% EtOH for 30 s;
- nuclease-free 50% EtOH for 30 s;
- 1/3-strength Shandon’s acidified hematoxylin for 30 s;
- rinse in nuclease-free H_2_O 1 min;
- rinse in freshly-prepared Scott’s tap water for 1 min;
- nuclease-free 50% EtOH for 30 s;
- nuclease-free 75% EtOH for 30 s;
- nuclease-free 95% EtOH for 30 s;
- nuclease-free 100% EtOH two times for 30 s;
- xylene two times for 30 s;
- the slides are then placed in xylene until ready for the LCM procedure.

**(B) Processing of slides for LCM**

**Figure 6:**
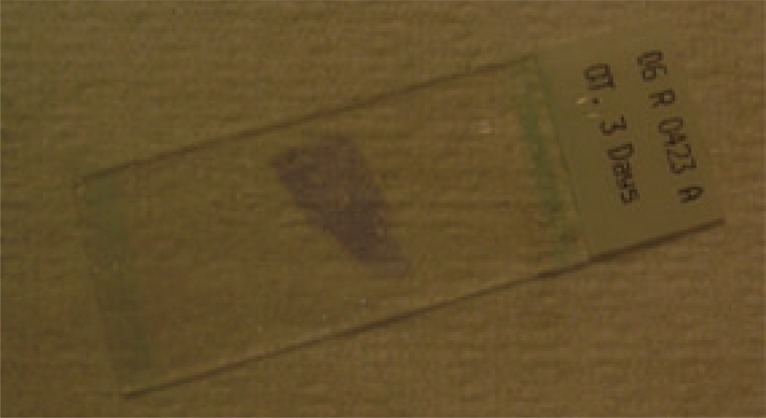
Hematoxylin-stained slide. This slide was demarcated with a PAP-pen (see greenish lines below and above tissue), stained with hematoxylin, and is now ready for dehydration preceding LCM.

Slides are allowed to dry (of xylene) 1 to 2 min before each laser cutting or laser capture event inside a dessicator. Slides are then ready for collection of cells (Fig. 6).

#### 4) Laser Cutting/Capture Microdissection (for 10 caps) (? TROUBLESHOOTING see Table 3)

**(A) Laser cutting**

**Figure 7:**
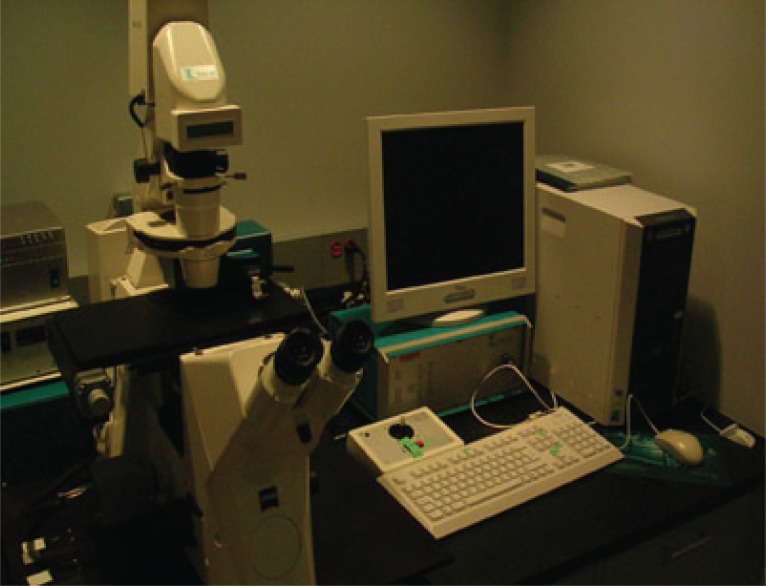
The Zeiss PALM apparatus. This instrument is used for the laser cutting (catapulting) procedure and requires a room with ample moisture so that random static discharges do not interfere with sample collection.

The PALM apparatus from Zeiss (Fig. 7) was used: ♦ **TIMING 2 h 30 min**

- Fill the cap of a sterile, RNase-free 200 μl PCR tube with 44 μl resuspension-lysis buffer part of the Invitrogen kit). Fit the cap on the tube holder of the PALM apparatus;
- Place the slide on stage of the microscope, visualize and circle cells of interest. The goal here is to collect 25-500 macrophages per cap. For our purposes, we collected 500 cells per sample in order to have enough material for standard curves and to assess each sample individually (by qPCR) for the presence of 8 different transcripts/targets of interest. **! CAUTION** Depending on animal species, age and type of cells isolated, the diameter of individual cells within a homogeneous cell population can differ. Macrophages are a good example (14). The PALM scope calculates the entire surface area collected, and this information can be used along with known average cell sizes to calculate the total number of cells collected. In some cases, the number of cells collected can be counted directly based on morphology and/or staining alone;
- Enable laser and set laser parameters (e.g. 15 μm laser spot size, 70 mW power, and 3.0 ms duration);
- Catapult cells of interest into the cap of the PCR tube and proceed immediately with the “Laser cutting RNA extraction procedure” (see below).

**(B) Laser capture**

**Figure 8:**
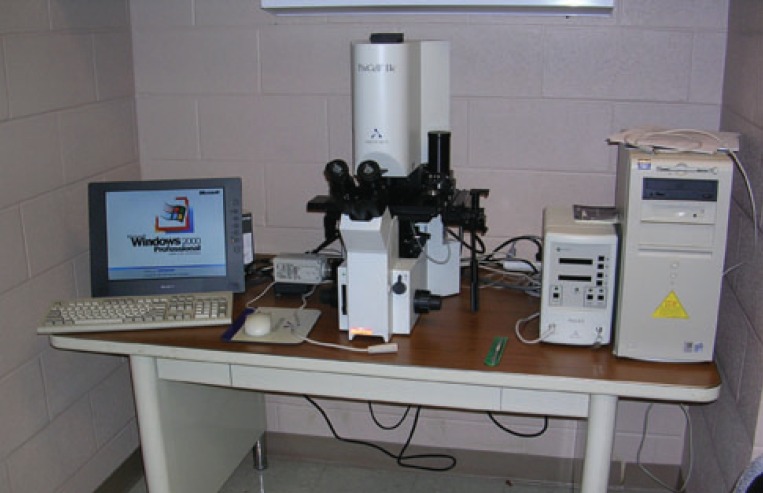
The Arcturus PixCell IIe apparatus. This instrument is used for the laser capture procedure and requires a room with low moisture so that tissues remain amply dry during cell collections.

The PixCell IIe Laser Capture Microscope (Arcturus; now Molecular Devices; Fig. 8) was used: ♦ **TIMING 3 h 30 min**

**Figure 9:**
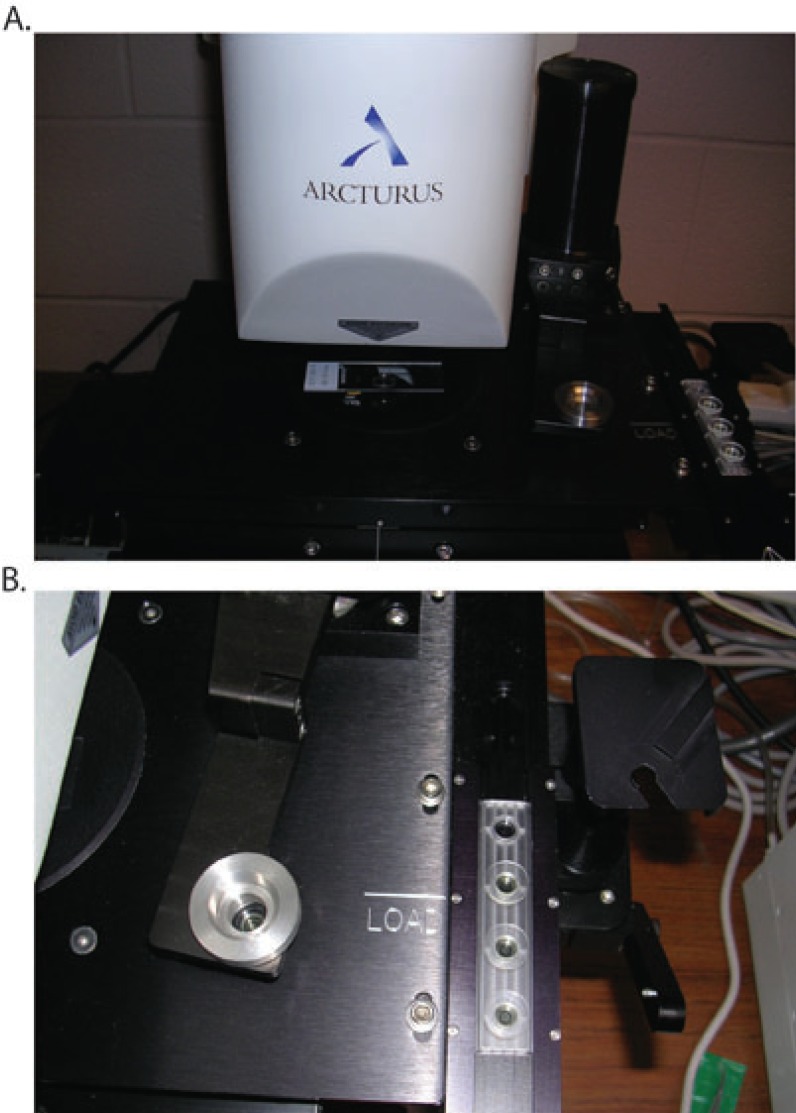
Positioning of slide and HS caps on the Arcturus Pix-Cell IIe stage. A, Frontal view of correct positioning of subject slide and HS caps; B, Top view of appropriate alignment of cap strip for loading of caps.

**Figure 10:**
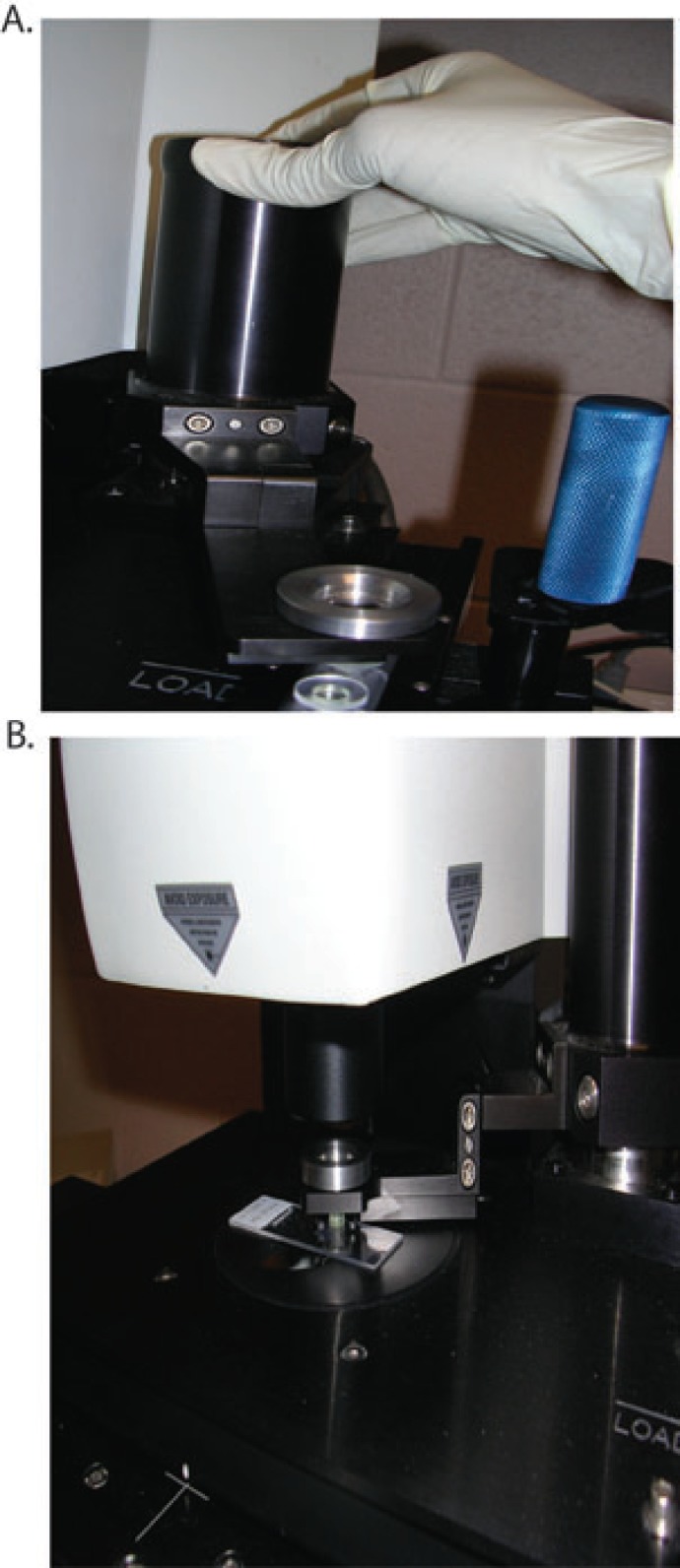
Picking up and moving HS caps into position. A, The rotating handle fitted with a cap holder is used to pick an HS cap from the cap strip; B, The rotating handle is gently swung over the subject slide and the HS cap is lowered down onto the tissue.

**Figure 11:**
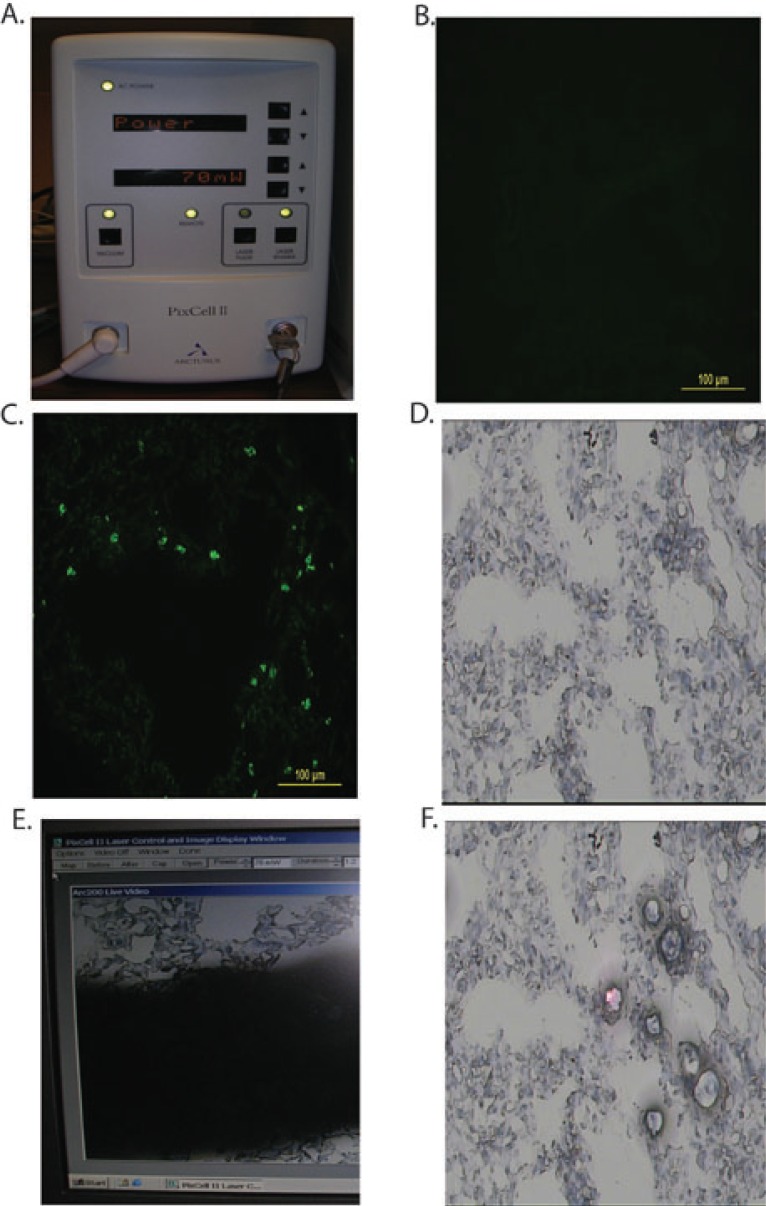
Laser enabling, cell visualization and collection. A. The laser power supply and slide vacuum are turned on; B, Example of a negative control for IF staining in which primary antibody was omitted; C, Example of IF staining of macrophages in lung sections (CD11b positive cells in this case); D, Hematoxylin staining of same tissue section. E. The circular boundary line on each HS cap (near the center of the capture polymer) can be seen on the computer screen, appearing as a thick black arc. F. Examples of laser shots exacted on a region of tissue showing the characteristic look of the melted capture polymer.

**Figure 12:**
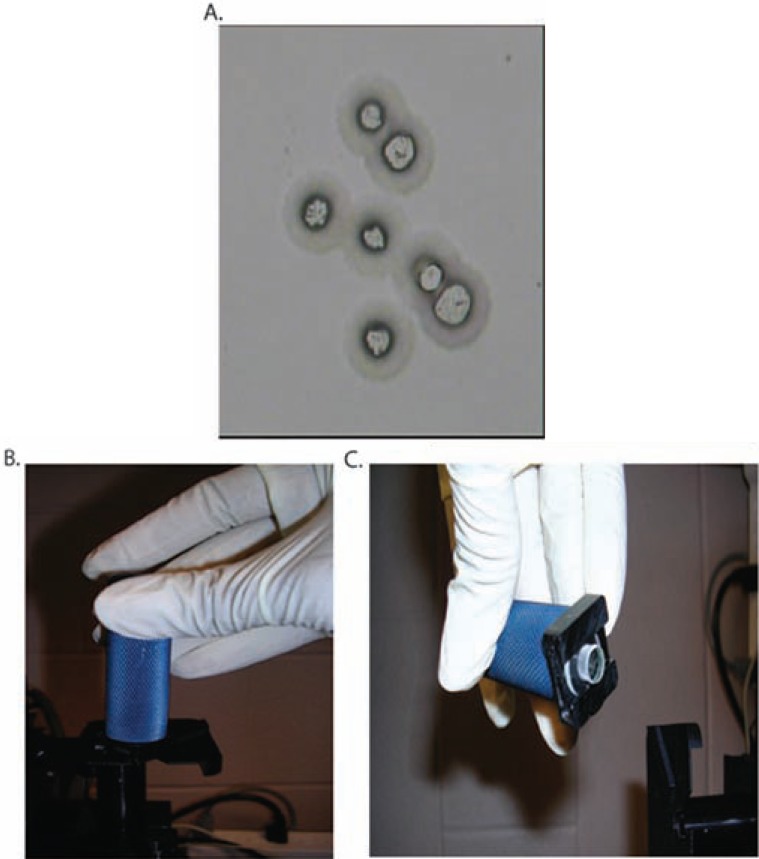
Proof of cell capture and retrieval of HS cap. A, Cells collected on the HS cap can be visualized directly under the LCM scope; B, Cap removal device is fitted onto the HS cap; C, HS cap is removed and made ready for RNA extraction.

- Place the slide on stage of the microscope and load HS caps in cap holder located on the right side of the stage. Make sure that the cap to be used is well aligned with the “LOAD” sign (Fig. 9A and Fig. 9B);
- Locate cells of interest on the slide by moving the joystick. Once cells are visualized, position the joystick in a perpendicular fashion to the table holding the microscope and turn on the vacuum to keep slide in a stable position;
- Lift one HS cap and position it on top of stabilized slide **! CAUTION** Be careful to avoid dragging the cap over the surface of the slide. Instead, lift the handle holding the HS cap up, rotate to the left towards the slide, and once the handle cannot turn further, gently lower handle down toward the slide (Fig. 10A and Fig. 10B);
- Enable laser and set laser parameters (e.g. 15 μm laser spot size, 70 mW power, and 3.0 ms duration; Fig. 11A);
- First direct laser to a region devoid of cells. Since no cells are collected during practice, there is no need to count the number of laser shots at this stage;
- Practice shooting to see if the capture polymer on the surface of the HS cap touching the slide is melting (laser shot should be of a desired size, with a black ring surrounding a white surface). Note: the laser spot size can be adjusted to capture 1 or multiple cells and the user needs to be cognizant of this and the efficacy at which the laser is doing its job at melting the intended targets to the cap surface polymer. **! CAUTION** Humidity of the subject slides is the major concern when collection is difficult (? TROUBLESHOOTING see Table 3);
- Redirect the field of vision to cells of interest (Fig. 11B, Fig. 11C and Fig. 11D), and make sure that the target cells appear on the inside of the black circle on the polymer on the cap (on the screen, a collection boundary line is visible on the surface of the capture polymer on the cap – which appears as a dark black circle, Fig. 11E). Note: Although Molecular Devices suggests staying within this black circle for cell collection (due to the CapSure assembly they suggest using), our “Polymer-Peel Lysis Method”^13^ (see also the Invitrogen user manual for CellsDirect™ One-Step qRT-PCR Kits, Version B, *25-0870*) allows for collection of cells anywhere on the cap. Adjust the spot size of the laser and fire repeatedly when necessary until the desired cells are melted to the capture polymer (Fig. 11F). Note: laser spot size can be changed to capture 1 or multiple cells (? TROUBLESHOOTING see Table 3);
- Once all cells are collected, gently lift the handle carrying the HS cap from slide, rotate towards the right side of the stage:

- Disable vacuum, and remove slide;
- In order to ensure that cells have been collected in the cap, the handle holding the cap can be gently rotated back towards the objective, the field of vision can be adjusted, and cells captured on the cap can be visualized (Fig. 12A);
- Next, the handle can be rotated back towards the right of the stage, and the cap can be removed from the handle (Fig. 12B and Fig. 12C);
- Immediately peel polymer tab from cap, carefully push to the bottom of the 0.5 ml microfuge tube containing resuspension-lysis buffer, close, and keep on ice until all samples are ready for the “Laser capture RNA extraction procedure” (see below) **♦ PAUSE POINT** At this point, all samples are considered to be in a neutral state as the lysis buffer contains chaotropic agents such as guanidinium isothiocyanate (GIT) which denatures nucleases while preserving nucleic acids. Although the procedure for RNA extraction can proceed the next day, we strongly recommend continuing with the RNA extraction procedure within 2 h after polymer immersion into lysis buffer.

#### 5) RNA extraction procedure (? TROUBLESHOOTING see Table 3)

**(A) Laser Cutting RNA isolation (for 10 caps) ♦ TIMING 1 h 10 min**

**Figure 13:**
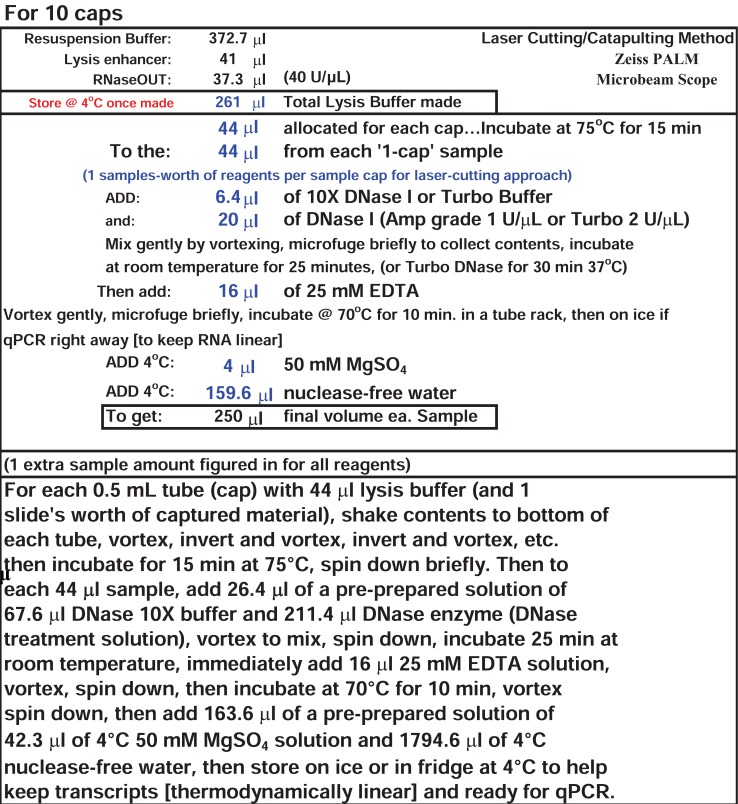
Laser cutting RNA extraction method.

Note: most reagents used in this procedure can be found in the CellsDirect™ kit. See Figure 13 for procedure summary:

- For each 0.5 ml Zeiss PALM tube cap with 44 μl lysis buffer (and 1 slide's worth of captured material), shake contents of lid to bottom of each tube, vortex and invert several times, then incubate for 15 min at 75°C and spin down briefly;
- Then to each 44 μl sample, add 26.4 μl of a pre-prepared solution of 67.6 μl DNase 10X buffer and 211.4 μl DNase enzyme (DNase treatment solution), vortex to mix, spin down, incubate 25 min at room temperature;
- Immediately add 16 μl 25 mM EDTA solution, vortex, spin down, then incubate at 70°C for 10 min;
- Vortex spin down, then add 164 μl of a pre-prepared solution of 42.3 μl of 4°C 50 mM MgSO_4_ solution and 1795 μl (of 4°C nuclease-free H_2_O). Keep on ice to help keep transcripts [thermodynamically linear] if performing qPCR right away. However, if qPCR will be performed at a later time, it is recommended to store the RNA at -80°C and avoid repeated freeze-thawing **♦ PAUSE POINT** We have tested RNA samples (by qPCR) prepared this way up to 3 months after their extraction (during which they were stored at 4°C), and have found no appreciable loss in signal; Ct values were identical to the first time we performed qPCR on the samples.

**(B) Laser Capture RNA isolation (for 10 caps) ♦ TIMING 1 h 10 min**

**Figure 14:**
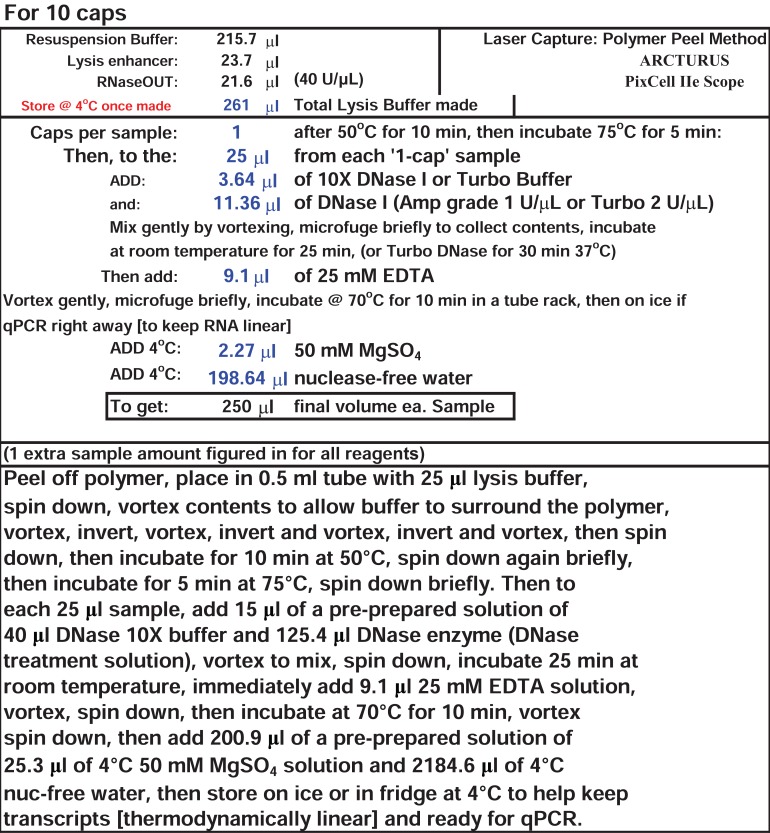
Laser capture RNA extraction method.

Note: most reagents used in this procedure can be found in the CellsDirect™ kit. See Figure 14 for procedure summary:

- Peel off polymer, place in 0.5 ml tube with 25 μl resuspension-lysis buffer, spin down, vortex contents to allow buffer to surround the polymer (optional: agitate and “stab at”) polymer tab with a 100 μl pipet and take up and re-dispense the resuspension-lysis buffer in and around the tab as much as possible for about 30 s);
- Vortex and spin down three times, then incubate for 10 min at 50°C;
- Spin down again briefly, incubate for 5 min at 75°C, and spin down briefly;
- Then, to each 25 μl sample, add 15 μl of a pre-prepared solution of 40 μl DNase 10X buffer and 125.4 μl DNase enzyme (DNase treatment solution), vortex to mix, spin down, incubate 25 min at room temperature;
- Immediately add 9.1 μl 25 mM EDTA solution, vortex, spin down, then incubate at 70°C for 10 min;
- Vortex spin down, then add 201 μl of a pre-prepared solution of 25.3 μl of 4°C 50 mM MgSO_4_ solution and 2185 μl (of 4°C nuclease-free H_2_O). Keep on ice to help keep transcripts [thermodynamically linear] if performing qPCR right away. However, if qPCR will be performed at a later time, it is recommended to store the RNA a -80°C and avoid repeated freeze-thawing **♦ PAUSE POINT** We have tested RNA samples (by qPCR) prepared this way up to 3 months after their extraction (during which they were stored at 4°C), and have found no appreciable loss in signal; Ct values were identical to the first time we performed qPCR on the samples.

#### 6) qPCR procedure (per plate per target) (? TROUBLESHOOTING see Table 3)

The PREXCEL-Q-based LCM approach was used (6, 7).

**(A) 1 day prior to qPCR: Labeling of tubes, master mix preparation, machine programming ♦ TIMING 1 h**

**Figure 15:**
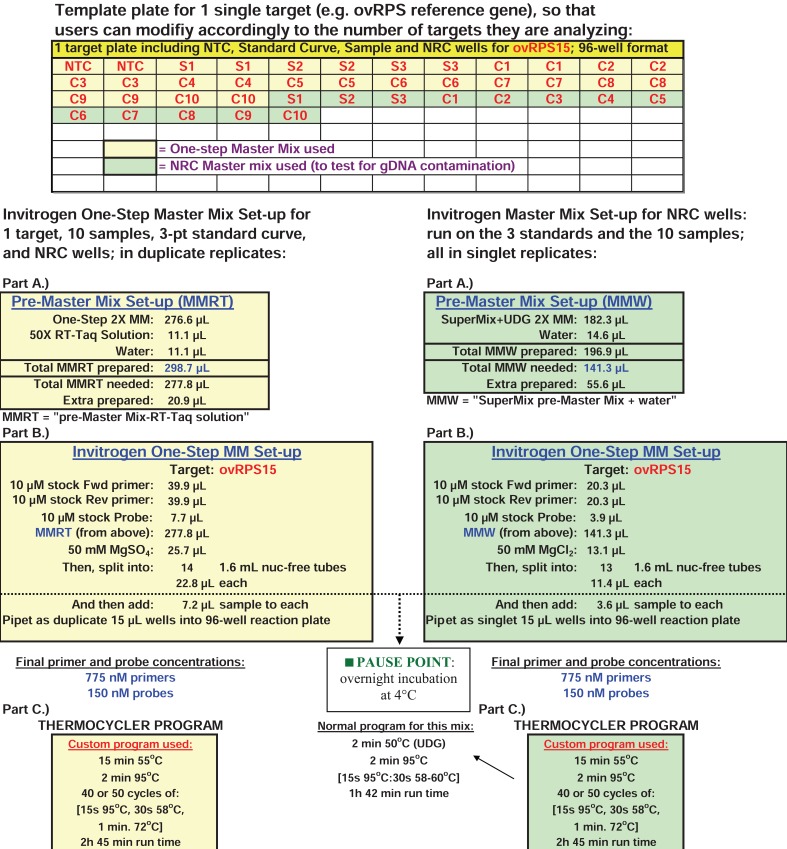
Depiction of set-up requirements for 1-target, 10 experimental LCM-RNA isolates already diluted to standard curve mid-range, 3-point serial 1:2 standard curve, NTC wells and NRC wells.

- LCM coupled with qPCR can be a lengthy procedure. We recommend making the master mixes and aliquotting in to microfuge tubes 1 day prior to LCM. A template master mix and plate setup for the assessment of 1 target gene in 10 samples can be found in Figure 15;
- Primers are all used at 775 nM and fluorogenic Taq-Man probes are used at 150 nM in the final qPCR reactions;
- The final Mg^+2^ needs to be adjusted to 5.5 mM in all final reactions. **♦ PAUSE POINT** We have found that the one-step master mix we use is stable overnight at 4°C when already pre-dispensed into nuclease-free microfuge tubes.

**(B) Day of qPCR ♦ TIMING 3 h**

- Following collection of samples and RNA isolation, standards were generated using an equal mixture of RNA samples used in the study that positively express all targets of interest for the qPCR study at hand. Such a mixture is referred to as “Stock I”;
- In order to generate a 3-point standard curve, standards obtained from Stock I were serially diluted 1:2;
- Samples were also diluted to the calculated standard curve midpoint ng/μl in-well concentration for all reactions;
- Next, using the master mixes pre-aliquotted in microfuge tubes (see step A and Figure 15), 3.6 μl sample (H_2_O, standard or RNA) is added per each 15 μl total qPCR reaction;
- Run plate (s) on the thermocycler using conditions listed in Figure 15.

**Table 3:** Troubleshooting

Problem	Possible Reason	Solution

**Procurement of tissue**		
RNA integrity compromised	Original tissue not frozen fast enough (liquid N2)	Take utmost care with tissue collection and handling – be aware of all possible sources of RNA degradation (temperature, environmental RNases). Use RNase-free tubes, tips and proper PPE
	Repeated freeze-thawing of tissues before RNA isolation	
	Improper handling of tissues (without gloves), introduction of RNases	
Cells not collecting or lifting properly during LCM	Tissues microtomed too thickly	Try sectioning at 5 μm
Tissues fall off of slides	Slide type used and/or slide coating used Pipetting too aggressively onto tissue	Trial and error – depending on what slides are used; can cut slides in duplicate; when administering reagents to slides, practice gentle pipetting
**Immunofluorescence**		
Background too high	Blocking and buffer system not appropriate; antibody concentration(s) too high; antibody binding nonspecifically; choice of fixative incorrect	Try TRIS-based buffer system at pH 7.6; try different blockers; try rinsing for 30 minutes after blocker application; try a dilution range of the antibodies used; order different antibody; try acetone or other type of fixative (if ethanol fails)
No staining	Antibody not specific enough	Try a different antibody
**Hematoxylin staining**		
Cells too dark, hard to visualize	Hematoxylin procedure not performed correctly	Shorter exposure to hematoxylin (e.g. 30 s); or longer exposure (e.g. 1 min)
Cells too light, hard to visualize		
**LCM**		
Laser works intermittently	Age of laser	Replace laser soon
Cells not lifting correctly by laser capture	Humidity of the LCM room too high	Dip slides back into xylene or place in dessicator, then re-dry under a fume hood and try again; try flattening tissue onto slide using a Prep Strip™
Cells not catapulting correctly by laser cutting	Humidity of the LCM room too low – static charges interfering	Add humidity control capability to the room in which scope resides
Unintended cells collected in addition to target cells	Diameter of laser, power of laser, duration of laser pulsing, humidity of tissue	Adjust laser size and other scope settings/parameters appropriately
**RNA extraction**		
Low RNA integrity and poor yield	Not incubating LCM samples at the specified temperatures per RNA extraction process; buffers prepared or used incorrectly; not enough cells collected by LCM; samples diluted too far to be useful in qPCR	Be mindful of each step and perform it with care and precision; prepare buffers fresh – preferably the dayof; collect more cells per sample; use P-Q more precisely to determine appropriate sample dilutions per cell type captured; take care and always be aware of and try to eliminate RNase contamination of samples at every step along the way
**qPCR**		
Standard curves do not look right	Low target amount; improper dilution of samples and standards; pipetting technique and/or pipettes are not calibrated; master mix not set-up correctly; machine error	Redesign assay to include different target(s) of interest; weigh water to pipet accuracy (i.e. 200 μl of H_2_O should weigh 0.2 grams at standard temperature and pressure); work on pipetting technique; do not always trust electronic pipets (check them too); recheck master mix setup; check machine bulb, power supply and any error messages
No amplification at all	Target not expressed in samples of interest; no cells collected; severe problem with setup and/or original tissue procurement(s); machine error	Redesign assay to include a different target or targets of interest; re-do LCM and make visual confirmation of collected cells; redesign assay and/or re-check calculations if not using PQ; check machine bulb, power supply and any error messages
Sample, standard and NRC wells show similar Ct values	DNase treatment did not work	DNase treat samples again if original isolates are still available; otherwise re-do LCM and be sure reagents are fresh. Amplification Grade DNase I is very temperature sensitive – be sure to store it appropriately when not in use
NTC wells show amplification	Unintentional introduction of sample material into NTC wells; templatecontaminated dust in the qPCR room settling into wells during setup; primer dimer formation(s) (if using SYBR Green-based qPCR)	Treat the qPCR room, all working surfaces and pipets with 10% bleach; remove dust; use new pipet tips; use proper PPE; redesign primers/probe; use fresh reagents

## ANTICIPATED RESULTS

**Figure 16:**
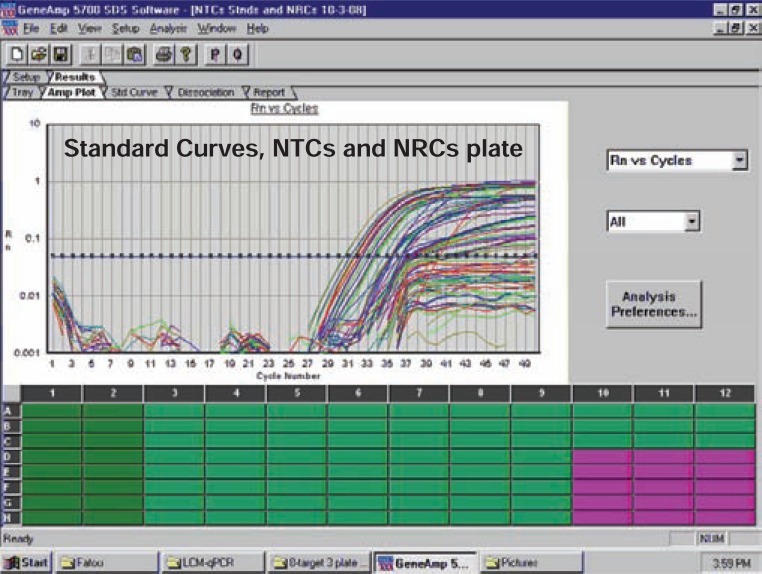
Example of LCM-qPCR sample amplifications for a plate containing standard curves, NTC wells and NRC wells for 8 targets of interest.

**Figure 17:**
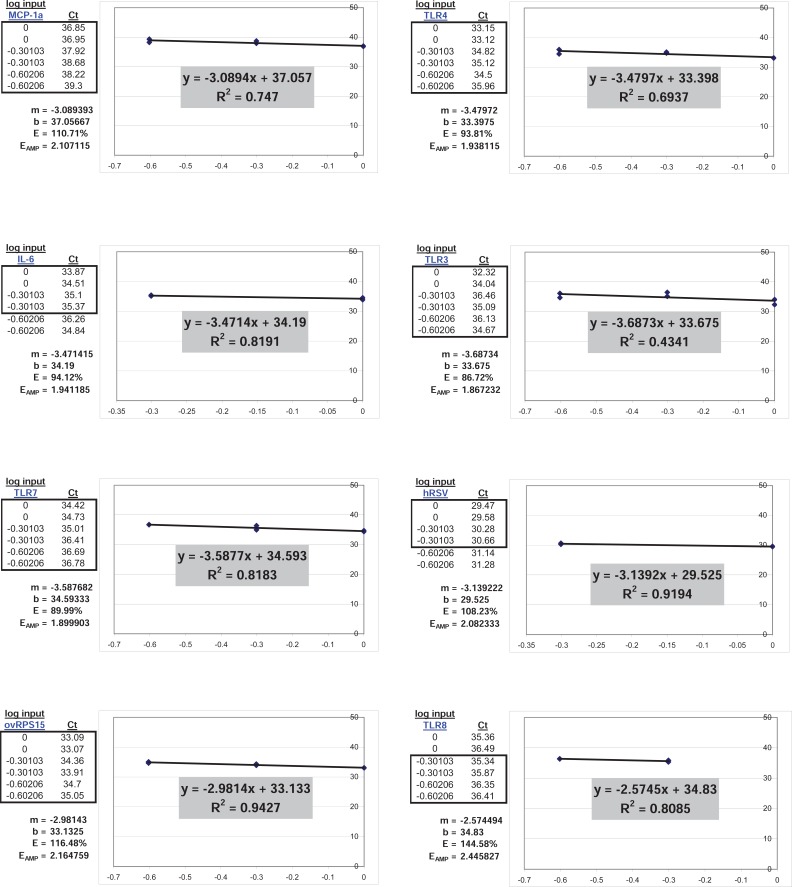
Standard curves generated for 8 LCM-qPCR targets: MCP-1, TLR4, IL-6, TLR3, TLR7, hRSV, ovRPS15 and TLR8.

Even though LCM collects miniscule amounts of material, it is possible for users to see collected cells on the collection cap (which look like scratch marks to the naked eye, or like actual cells under the microscope), or inside the resuspension-lysis buffer inside the lid (for laser cutting) by microscopic inspection. For 500 macrophages collected, using 10 pg of total RNA per macrophage, we obtained an approximate yield of 5 ng of total RNA. Each RNA sample was brought up into a final volume of 250 μl which provided enough material, from 10 such samples, to assess 8 targets of interest by qPCR (replete with 3-point standard curves, individual sample assessments – all in duplicate, and NRC wells for genomic DNA analysis, see Figure 16 and Figure 17). Abundantly expressed target transcripts are typically able to generate better standard curves than rarely expressed targets. For example, of the 8 targets assessed, MCP-1 (15) was among 7 targets which were able to generate reasonable standard curves, with very acceptable amplification efficiencies, whereas TLR8 did not. We reasoned that, since the other 7 targets behaved nicely, RNA integrity was not the problem. This suggests that TLR8 is simply a rare target in our samples. When DNase treating the RNA samples with reagents not included in the CellsDirect™ One-Step qRT-PCR Kit with ROX, one can expect varying degrees of success. For example, we tested the kit’s Amplification Grade DNase I in comparison to Ambion’s TURBO DNase I, and found that the kit’s Amplification Grade DNase I worked better in this application. When using IF to label specific cells to be collected by LCM, non-specific background fluorescence can make it difficult to identify the cells sought for study. When comparing expression of a cell-specific (mRNA) transcript from LCM-derived material to whole tissue-derived material, one can expect lower Ct values (thus higher expression) from the enriched LCM samples with or without normalization to an appropriate, stable reference gene (e.g. ovRPS15 in this study). Additional considerations for this section can be found elsewhere (3, 4).
